# A Mendelian randomization study of type 2 diabetes and cancer risk in East Asians

**DOI:** 10.1186/s12935-025-03929-1

**Published:** 2025-08-04

**Authors:** Ling Li, Fangxuan Li, Zhanyu Pan

**Affiliations:** 1 Department of Integrated Traditional and Western Medicine, Key Laboratory of Cancer Prevention and Therapy, Tianjin Medical University Cancer Institute and Hospital, National Clinical Research Center for Cancer, Tianjin’s Clinical Research Center for Cancer, Tianjin, 300060 China; 2 Department of Cancer Prevention, Tianjin Medical University Cancer Institute and Hospital, Tianjin, China

**Keywords:** Type 2 diabetes, Cancer, Mendelian randomization

## Abstract

Our research aims to explore genetic correlation between T2D predisposition and risks of several cancers, which have been predominantly focused on populations of European ancestry. In an East Asian population, we leverage two-sample Mendelian Randomization to investigate the complex association between Type 2 Diabetes (T2D) and cancer susceptibility. This investigation utilizes genetic data summarized from three reputable sources: the Japanese ENcyclopedia of GEnetic associations by Riken (JENGER), the Asian Genetic Epidemiology Network (AGEN), and the Meta Analyses of Glucose and Insulin-related traits (MAGIC). We explored the associations between exposure datasets, which included T2D, glycated hemoglobin (HbA1c) and fasting glucose (FG) levels, and the risk of several prevalent cancers for the outcome datasets. By analyzing 174 SNPs associated with T2D, 15 SNPs related to FG, and 74 SNPs linked to HbA1c, we discovered a significant inverse relationship between T2D and the majority of cancers, including gastric (OR = 0.875, 95%CI: 0.825–0.928), breast (OR = 0.907, 95%CI: 0.850–0.967), esophageal (OR = 0.761, 95%CI:0.681–0.851), colorectal (OR = 0.877, 95%CI:0.834–0.923), hematological malignancy (OR = 0.837, 95%CI:0.752–0.933), lung (OR = 0.916, 95%CI:0.858–0.977), hepatocellular (OR = 0.865, 95%CI:0.787–0.951), prostate (OR = 0.862, 95%CI:0.812–0.914), and endometrial cancer (OR = 0.841). The power of gastric cancer, breast cancer, esophageal cancer, colorectal cancer and prostate cancer reached the desired 80%. Conversely, we did not observe a reverse correlation between T2D and specific cancers in the East Asian population. There is limited evidence that genetically predicted FG and HbA1c levels are associated with specific cancer. Moreover, conducting multivariable MR analysis did not alter the correlation between T2D and specific tumors. These findings explain the causal associations of genetic liability to T2D with various types of cancers in East Asian ancestry, and this association is not affected by glycemic biomarkers.

**Supplementary Information:**

The online version contains supplementary material available at 10.1186/s12935-025-03929-1.

## Introduction

The prevalence of diabetes is on the rise globally, with a significant increase observed in Asia [1]. According to the 10th edition of the Diabetes Atlas, as reported on https://www.diabetes.org/, the number of adults living with diabetes is projected to reach 113 million by 2030 and escalate further to 151 million by 2045. While numerous genetic studies have established a causal link between Type 2 Diabetes (T2D) and the occurrence of certain tumors, focusing primarily on populations of European ancestry, research specifically targeting East Asian populations is notably lacking, especially concerning T2D [2–4]. Previous investigations have highlighted considerable interethnic differences in the frequency and impact of risk alleles, leading to distinct T2D characteristics in East Asians compared to Europeans, such as lower body mass index (BMI) and earlier onset age [5, 6]. Moreover, variations in the causal relationships between T2D and other diseases have also been observed between East Asian and European populations [7, 8]. Additionally, variations in the causal relationships between T2D and other diseases have been noted between these populations, with differing associations between various diseases and cancer among ethnic groups. This diversity is often attributed to the unique genetic architectures, which limit the applicability of genetic findings across populations. In response to the need for clarity on the T2D-cancer risk correlation specifically within the East Asian demographic, our study employs a two-sample Mendelian Randomization (MR) method.

It has been previously reported that the relationship between pancreatic cancer and diabetes is complex, characterized by bidirectionality and reciprocal causation [9]. In limited Asian studies, only digestive system and prostate tumors were investigated [10, 11]. Addressing this, we performed a bidirectional MR analysis on T2D and all cancers in East Asian population. This effort is part of a broader initiative to deepen the understanding of the T2D-cancer correlation in East Asian groups, including a comprehensive review of clinical literature pertaining to T2D and cancer within Asian populations.

## Material & methods

### Sources of data

A two-sample MR study was conducted using genetic data from Japanese ENcyclopedia of GEnetic associations by Riken (JENGER) [12], Asian Genetic Epidemiology Network (AGEN) [13], and Meta Analyses of Glucose and Insulin-related traits (MAGIC) [8]. For cancer GWAS (JENGER), we included 12 physician-verified cancer types (Table 1), with diagnoses based on ICD-10 codes and pathological confirmation. The analysis comprised 179,660 cases and 32,793 population controls of Japanese ancestry. Phase 3 of the 1000 Genomes Project (1KG Phase3) served as the reference panel during GWAS imputation [14]. All studies were approved by their respective ethical committees.

**Table 1:** Data sources of exposure and outcome

		Consortium	participants	Web source if publicly available
Exposure	T2D	Asian Genetic Epidrmiology Network (AGEN) consortium [10]	77,418 individuals with T2D and 356,122 healthy control individuals of East Asian ancestry	https://blog.nus.edu.sg/agen/summary-statistics/t2d-2020/
	Glycemic traits	Meta-Analyses of Glucose and Insulin-related traits Consortium(MAGIC)[7]	The sample sizes were 35,619 FG and 146,806 HbA1c in the analysis of East Asian ancestry.	http://magicinvestigators.org/
Outcome	Breast cancer	the Japanese ENcyclopedia of GEnetic associations by Riken (JENGER) consortium [9]	5,552 Breast cancer cases and 89,731 controls of East Asian ancestry.	http://jenger.riken.jp/en/
	Lung cancer		4,050 Lung cancer cases and 208,403 controls of East Asian ancestry	
	Esophageal cancer		1,300 Esophageal cancer cases and 195,745 controls of East Asian ancestry	
	Gastric cancer		6,563 Gastric cancer cases and 195,745 controls of East Asian ancestry	
	Pancreatic cancer		442 Pancreatic cancer cases and 195,745 controls of East Asian ancestry	
	Biliary tract cancer		339 Biliary tract cancer cases and 195,745 controls of East Asian ancestry	
	hepatocellular carcinoma		1,866 hepatocellular carcinoma cases and 195,745 controls of East Asian ancestry	
	Colorectal cancer		7,062 Colorectal cancer cases and 195,745 controls of East Asian ancestry	
	Endometrial cancer		999 Endometrial cancer cases and 89,731 controls of East Asian ancestry	
	Ovarian cancer		720 Ovarian cancer cases and 89,731 controls of East Asian ancestry	
	Cervical cancer		605 Cervical cancer cases and 89,731 controls of East Asian ancestry	
	Prostate cancer		5,408 Prostate cancer cases and 103,939 controls of East Asian ancestry	
	Hematological malignancy		1,236 Hematological malignancy cases and 211,217 controls of East Asian ancestry	

T2D: Type 2 diabetes; FG: fasting glucose; HbA1c: glycated hemoglobin

Exposure datasets include T2D and Glycemic traits, while outcome included datasets for several prevalent cancers including Breast cancer, Lung cancer, Esophageal cancer, Gastric cancer, Pancreatic cancer, Biliary tract cancer, hepatocellular carcinoma, Colorectal cancer, Endometrial cancer, Ovarian cancer, Cervical cancer, Prostate cancer, Hematological malignancy.

### Selection of instrumental variables

Based on a robust meta-analysis of 23 studies conducted by the Asian Genetic Epidemiology Network (AGEN), instrument variables for T2D were determined. This comprehensive analysis included a substantial sample size of 77,418 individuals with T2D and 356,122 healthy East Asian controls. The significant SNPs in our study explained up to 1.3% of the variance. To investigate the impact of glycemic traits on the causal relationship between T2D and cancers, instrument variables for glycated hemoglobin(HbA1c)and fasting glucose (FG) levels were determined. This comprehensive analysis included a substantial sample size of 35,619 individuals with FG and 146,806 individuals with HbA1c. All GWAS datasets employed the Phase 3 of the 1000 Genomes Project as the reference panel during the imputation stage.

We set the primary significance levels of indexed SNPs to 5 × 10^−8^ and r ^2^ to 0.001. Physical distance was set to 1 Mb. Instrumental variables with an F-statistic below 10 were considered weak and therefore were excluded from the analysis. Then independent SNPs that fulfill the above requirements were selected for this study. A total of 174 SNPs associated with T2D, 15 SNPs associated with FG, and 74 SNPs associated with HbA1c from AGEN and MAGIC were utilized for subsequent MR analysis. T2D, FG, and HbA1c instrumental variables are presented in Supplementary Table S1. Cancer instrumental variables are presented in Supplementary Table S2. Removed SNPs are presented in Supplementary Table S3.

### Statistical analysis

**Fig. 1:**
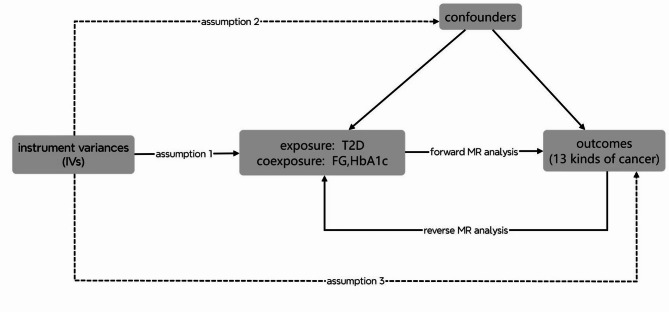
The study design for MR analysis between T2D and cancers

In our study, we rigorously adhered to the three instrumental variable (IV) assumptions essential for robust MR analysis. For IV assumption 1, which demands that IVs are associated with the exposure, we selected SNPs linked to the exposure trait. Addressing IV assumptions 2 and 3, which state that IVs should affect the outcome exclusively via the exposure and not be associated with any confounders, can be challenging due to the risks of horizontal pleiotropy and confounding factors. To navigate these challenges, we conducted extensive sensitivity analyses, including Cochran’s Q statistic to assess heterogeneity across SNPs and MR-Egger regression for detecting horizontal pleiotropy to evaluate the influence of individual SNPs. These methods helped us assess the validity of IV assumptions 2 and 3. Specifically, the MR-Egger regression intercept indicates horizontal pleiotropy, violating assumption 2 if significantly different from zero. The weighted median method can provide a reliable estimate even if only a majority of the IVs satisfy the assumptions. The use of a random-effects IVW model in cases of significant heterogeneity, indicated by Cochran’s Q statistic, helps address the potential pleiotropy among IVs. Moreover, the MR-PRESSO test detects outliers and horizontal pleiotropy. If outliers are identified, they should be excluded, followed by a reanalysis of the MR study. The final data, with outliers removed, will be presented in the figures and tables. The flowchart was shown in Fig. 1.

Previous research has indicated that in East Asian populations, the pathogenesis of T2D is associated with the methylation of TXNIP and CPT1A genes. This methylation process may be influenced by factors such as Fasting Glucose (FG) and Glycated Hemoglobin (HbA1c) [8]. To account for these influences in our study, we conducted a multivariable Inverse Variance Weighted (IVW)MR analysis to address assumption 2. The multivariable IVW approach allows for the simultaneous consideration of multiple exposure factors. This method helps mitigate the potential confounding impact of SNP-exposure correlations on indirect pathways to other assumed risk factors. It achieves this by incorporating summary genetic association data from both the exposure and risk factors into a weighted regression model, providing a more comprehensive assessment of risk. A significant impact on the outcome can be seen if the p-value is less than 0.05 after incorporating other exposure factors.

In our study, recognizing the critical importance of understanding bidirectional causality, we additionally performed a reverse MR analysis between T2D and various types of cancers. This reverse MR analysis was not just a mere extension of our primary research, but a vital step in comprehensively understanding the complex interplay between T2D and cancer. By investigating the possibility that genetic predispositions to T2D might influence the risk of developing cancer, and vice versa, this analysis aimed to uncover potential shared genetic pathways and reciprocal causative relationships. This approach is crucial as it moves beyond traditional unidirectional causality, allowing us to delve into the nuanced interdependencies that could have significant implications for both prevention and treatment strategies in T2D and cancer.

The results of MR estimates were shown as standard error (SE), P value (acquired for random effects IVW model), odds ratios (ORs), 95% confidence intervals (CIs), B, nSNP and removed SNP in Supplementary Table S4. Other results of MR estimates (acquired for fixed effects IVW model, horizontal pleiotropy, heterogeneity, and MR-PRESSO) are displayed as supplements in Supplementary Table S5. ORs and 95%CIs of cancer were scaled to one-unit increase in log odds of liability to T2D. The calculation of power for the analyses of T2D is performed on the website https://shiny.cnsgenomics.com/mRnd/. All statistical analyses were two-sided and performed by the package TwoSampleMR and MRPRESSO in Rstudio 4.2.1.

## Results

**Fig. 2:**
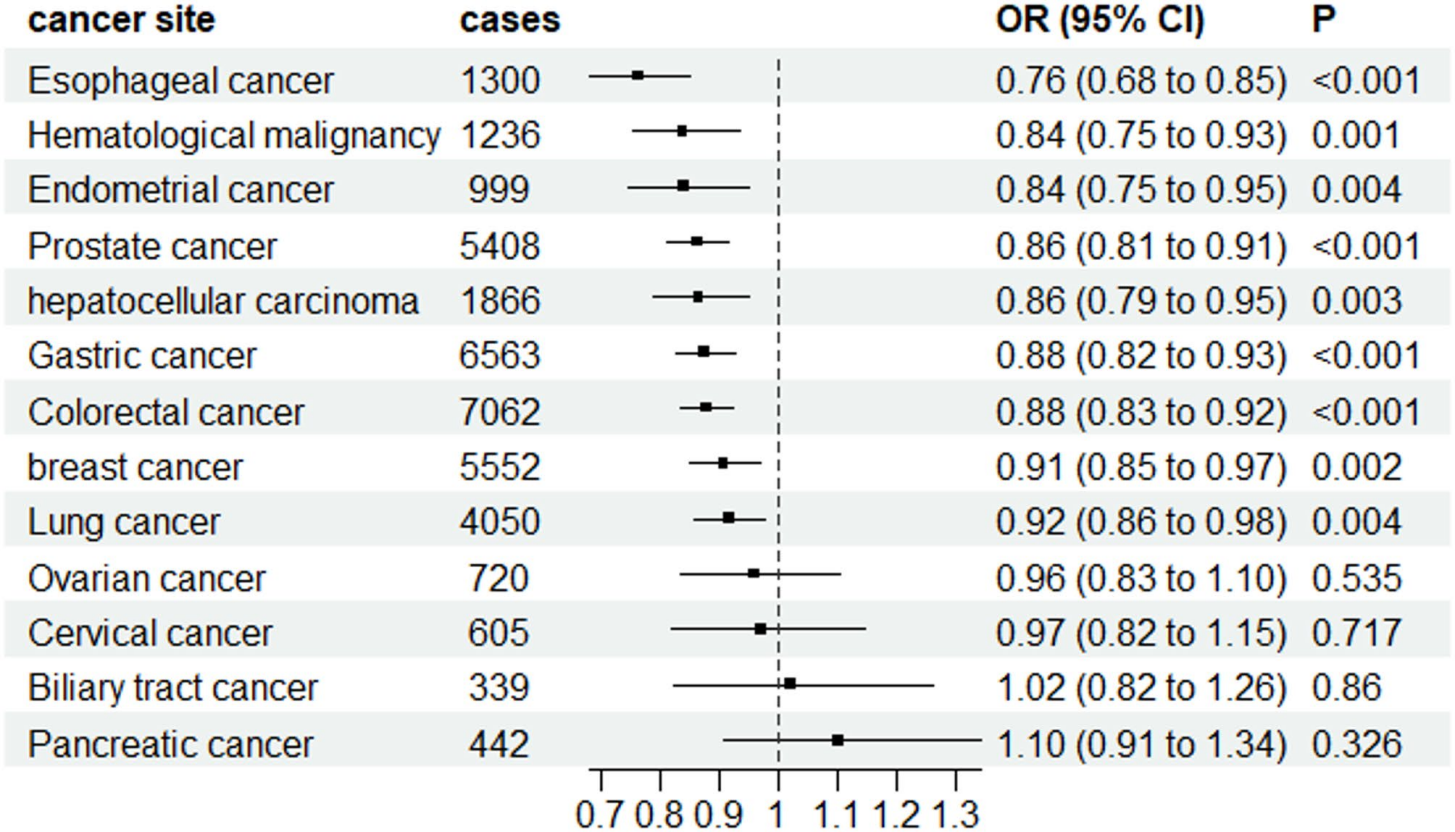
The results of MR estimate for associations between T2D and cancers using random-effects IVW mode

The forward MR analysis demonstrated a significant inverse genetic association between T2D and nine out of twelve cancer types, including gastric cancer, breast cancer, esophageal cancer, colorectal cancer, hematological malignancy, lung cancer, hepatocellular carcinoma, prostate cancer and endometrial cancer, as illustrated in Fig. 2. We detected statistical heterogeneity in the analysis of gastric cancer, breast cancer, esophageal cancer, lung cancer and prostate cancer. The MR-PRESSO test identified and removed the doutliers from them (rs9350271 and rs55734389 for gastric cancer, rs76878791 for breast cancer, rs1260326 for esophageal cancer, rs3869115 and rs9461650 for lung cancer, rs7501939 for prostate cancer). After excluding the outliers, we performed a revised MR analysis using the IVW model. The adjusted results are presented in supplementary Table S4. Interestingly, among the several common cancers investigated in our study, T2D showed a positive association with only two types of cancers: pancreatic cancer (OR = 1.103, 95%CI: 0.907–1.341) and biliary tract cancer (OR = 1.019, 95%CI: 0.823–1.263). However, the evidence for these associations was not conclusive as the p-values >0.05. Similarly, no statistical associations were found between T2D and the risk of ovarian and cervical cancer (Fig. 2). Table S5 presents the results obtained from five different MR analysis methods. Despite the discrepancies among the conclusions drawn by these methods, we have chosen to adopt the findings from the random effects IVW model as our primary research conclusion. This decision is based on the method’s high statistical efficiency and proven robustness, which make it a reliable choice for interpreting the causal relationship between the exposure and the outcome in our study. We also calculated the power of the MR analysis, which shown in Table 2. We found statistical power exceeded 80% for gastric cancer, breast cancer, esophageal cancer, colorectal cancer and prostate cancer.

**Table 2:** Power calculation in the analysis of MR associations betweenT2D and the risk of cancer https://shiny.cnsgenomics.com/mrnd/

exposure	outcome	sample size	power	F-statistic
T2D	Gastric cancer	202,308	1	42617.94
T2D	breast cancer	95,283	0.86	20072.72
T2D	Esophageal cancer	197,045	0.93	41509.27
T2D	Colorectal cancer	202,807	0.99	42723.06
T2D	Hematological malignancy	212,453	0.68	44755.02
T2D	Lung cancer	212,453	0.69	44755.02
T2D	hepatocellular carcinoma	197,611	0.68	41628.5
T2D	Pancreatic cancer	196,187	0.15	41328.53
T2D	Biliary tract cancer	196,084	0.05	41306.83
T2D	Prostate cancer	109,347	1	23035.36
T2D	Endometrial cancer	90,730	0.55	19113.62
T2D	Ovarian cancer	90,451	0.07	19054.84
T2D	Cervical cancer	90,336	0.06	19030.62

Although we found a statistically significant negative correlation between liver cancer, pancreatic cancer, prostate cancer, and cervical cancer with the occurrence of T2D in our reverse MR analysis, shown in Supplementary table S6. We also observed that the number of extracted SNPs was limited (shown in Supplementary Table S7). While 20 SNPs were identified for prostate cancer, their heterogeneity was noted, and no significant outliers were identified through MR-PRESSO analysis. After excluding the outliers, we still did not discover any meaningful correlations.

**Fig. 3:**
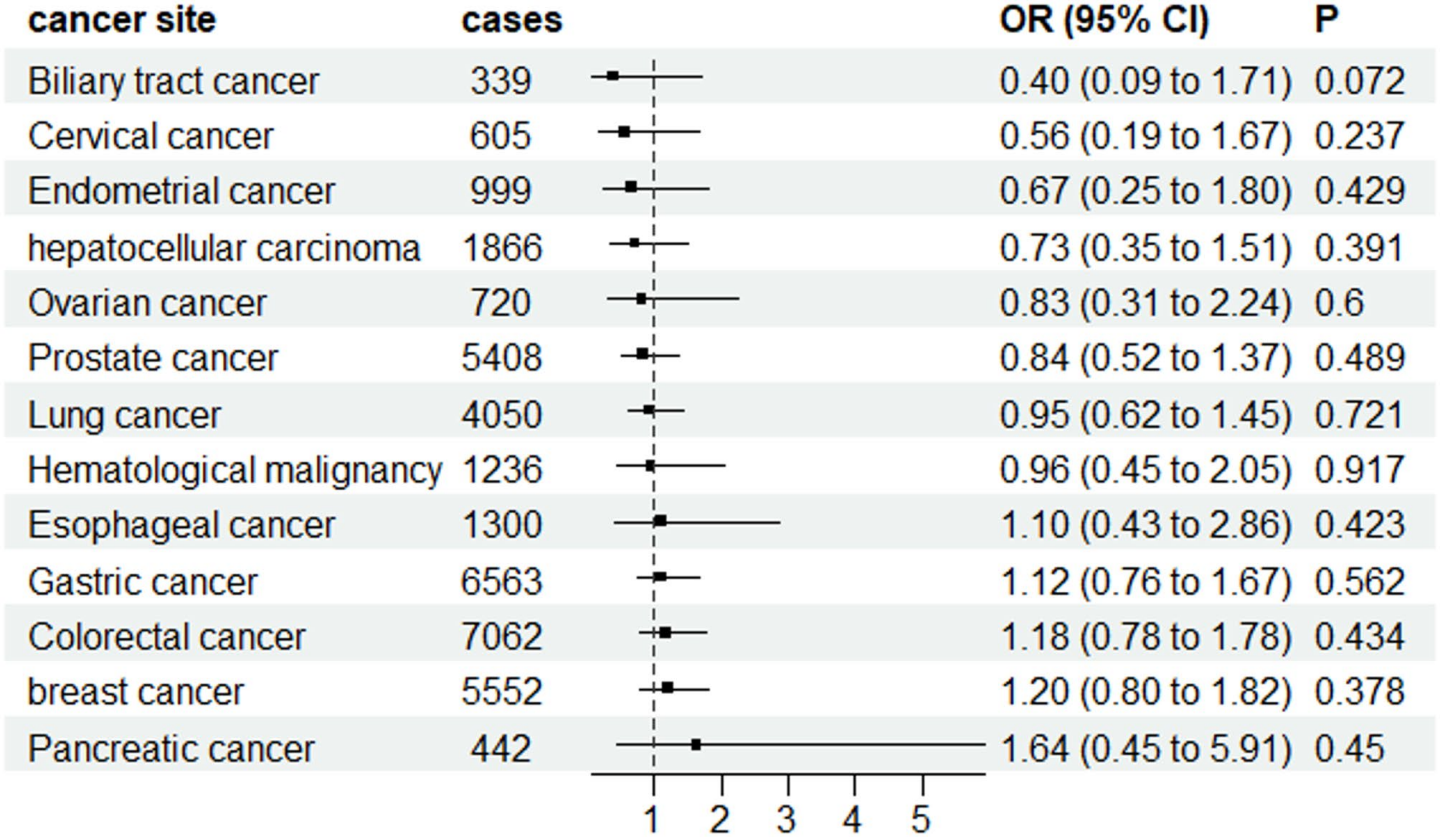
The results of MR estimate for associations between FG and cancers using random-effects IVW mode

**Fig. 4:**
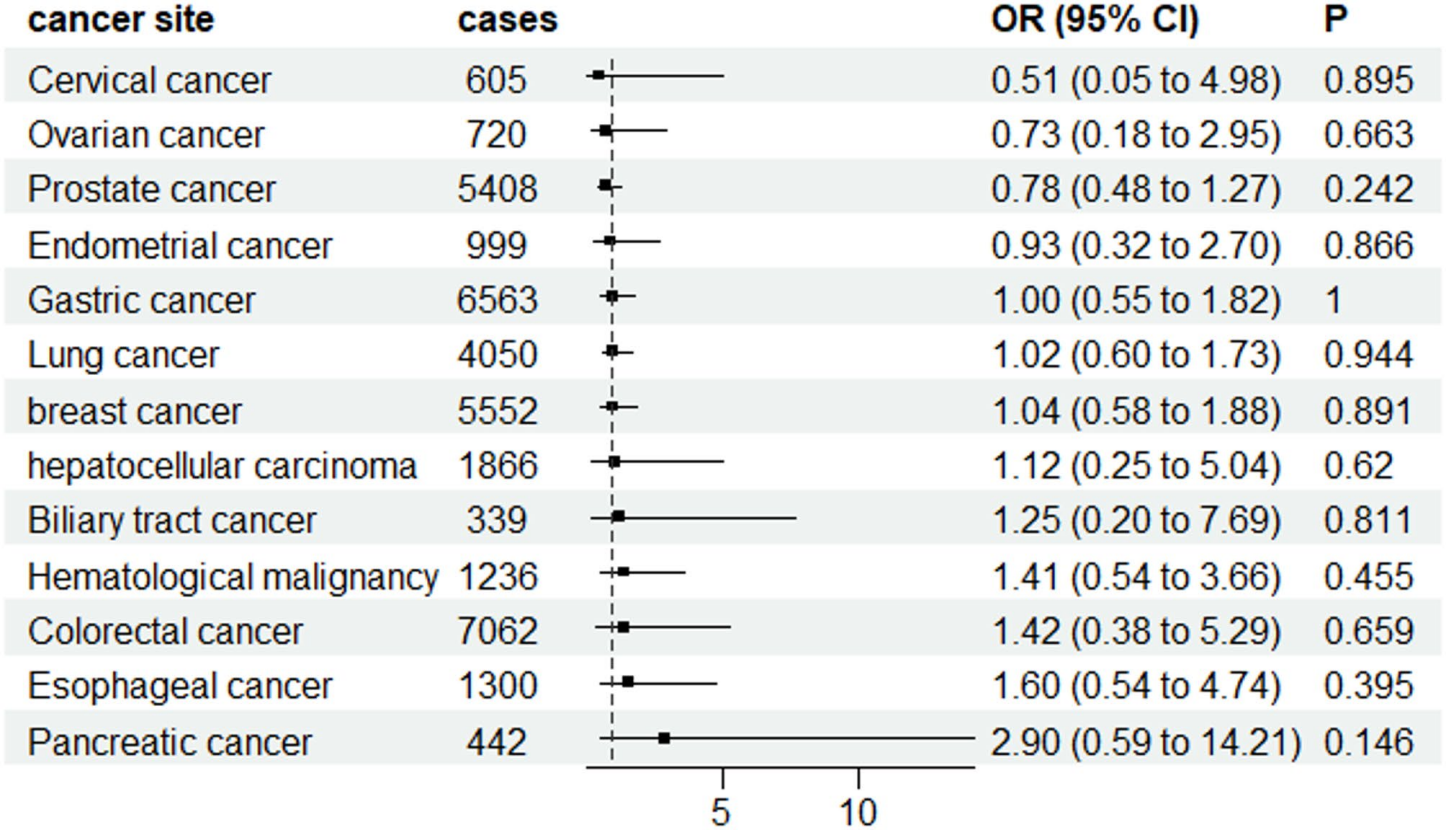
The results of MR estimate for associations between HbA1c and cancers using random-effects IVW mode

**Fig. 5:**
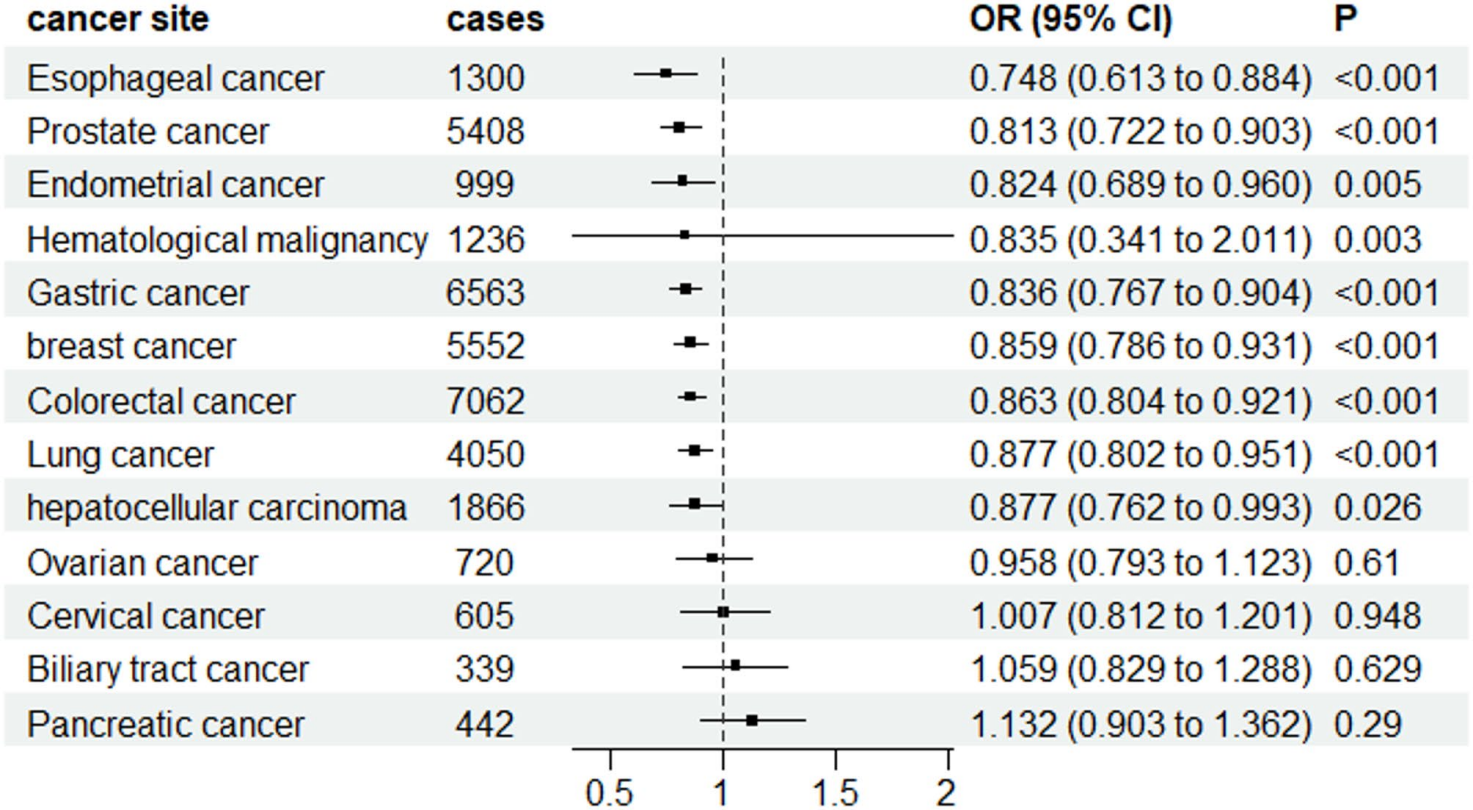
The results of multivariable IVW MR analysis for associations between T2D and cancers

The available evidence regarding the association between genetically predicted FG and HbA1c levels and cancer is limited (Figs. 3 and 4). After excluding the outliers, the results didn’t change significantly. The magnitude of the estimates was relatively strong for some cancer sites, although the precision was low in most analyses. We found the ORs were above 1.5 for genetically predicted high FG levels in relation to pancreatic cancer and below 0.5 for Biliary tract cancer (Fig. 3). For HbA1c levels, the ORs were above 1.5 for Pancreatic and Esophageal cancer (Fig. 4). This putative association between T2D and the risk of cancers was not influenced by FG, or HbA1c initiation according to multivariable MR analysis (Fig. 5). The results of multivariable IVW MR analysis are shown in Supplementary Table S8.

## Discussion

Our study is a MR study that systematically explores the causal associations of genetic liability to T2D with various types of cancers in East Asian ancestry. We found genetic evidence that liability to T2D was associated with decreased risks of many cancers include gastric cancer, breast cancer, esophageal cancer, colorectal cancer, hematological malignancy, lung cancer, prostate cancer and endometrial cancer. The underlying biological mechanism behind this relationship may be the evolutionary pressure of metabolic pathway selection. For example, MTNR1B rs10830963 and HNF1B rs757210, as T2D-related genes, have inhibitory effects on prostate cancer [15, 16]. Adiponectin is closely related to the occurrence of T2D, but it is a protective factor for the occurrence of breast cancer [17]. Another potential biological mechanism may be the sex-steroid axis modulation. For example, T2D-related hyperinsulinemia increases the concentration of SHBG, depletes the bioavailable estrogen in breast tissue, and reduces the risk of breast cancer [18].

Our study presents novel findings that diverge from previous reports on individuals of European ancestry [4]. Yuan et al. carried out a two-sample MR analysis of T2D and common tumorigenesis risks in European species [4]. The results reveal a correlation between T2D genetic predisposition and a higher likelihood of developing pancreatic, kidney, uterine, and cervical cancer. The variations observed in MR results between East Asian and European populations can likely be attributed to interethnic disparities in the pathophysiology and epidemiology ofT2D [19]: First, compared to European populations, East Asian individuals demonstrate a characteristic predisposition to develop T2D at relatively lower body mass index (BMI) thresholds [20]. And many literatures report a positive dose-response relationship between BMI and cancer risk [21, 22]. Secondly, East Asian patients often experience the onset of T2D at a younger age in comparison to European patients [19]. This accelerated disease progression consequently leads to earlier therapeutic intervention with glucose-lowering agents demonstrating potential oncostatic effects, including metformin and sulfonylurea compounds, within East Asian populations [23–25]. These interethnic distinctions may offer insights into the discordant MR findings observed in East Asian and European populations. Worth noting is a MR study based on data from the UK Biobank, which analyzed the association between T2D and cancer occurrence in the South Asian ancestry [26]. They found a decreased risk for cancer mortality (HR: 0.59, 95% CI 0.41 to 0.85) and incidence (HR: 0.80, 95% CI 0.70 to 0.92), further supporting the notion that T2D-related genes in the Asian ancestry differ from those in the European population and may exert inhibitory effects on cancer development.

In our MR analysis, FG and HbA1c levels had not found measurable causal effects on cancer occurrence. Furthermore, multivariable IVW MR adjustments confirmed that these glycemic biomarkers did not modify the established association between T2D and cancer incidence. The independence from FG and HbA1c implies potential non-glycemic pathways may mediate the correlation between T2D predisposition and decreased risks of several cancers.

Several MR analyses from European ancestry have demonstrated a causal relationship between diabetes and pancreatic cancer incidence [27]. However, there have also been studies reporting no significant association between them by Robert [28]. A population-based prospective study conducted on Asian populations also yielded results consistent with the findings of the Robert study [29]. Additionally, our study results provide additional evidence suggesting that T2D may not be the sole factor contributing to the observed correlation between T2D and the increased risk of pancreatic cancer. Interestingly, reverse MR analysis suggested a potential protective effect of pancreatic cancer liability on T2D development. However, this observation should be interpreted with caution due to instrumental variable limitations, as only a single genome-wide significant SNP (rs60579835) was available for analysis, potentially introducing weak instrument bias and compromising the reliability of causal estimates.

Real-world observational studies and meta-analysis results support that T2D is associated with increased cancer risk [18–27, 30–40], which wasn’t in line with our research conclusions. It may be due to the fact that genetic proxies represent lifelong exposure whereas clinical diabetes typically manifests in midlife, creating temporal mismatch in effect estimation. Understanding how genetic predisposition to T2D influences cancer risk differently in these populations can provide valuable insights into the complex interplay between these diseases. Investigating the distinct biological pathways and genetic factors involved in T2D-cancer associations among East Asian and European populations may pave the way for precision prevention strategies tailored to specific ancestral backgrounds, ultimately mitigating the risk of these diseases more effectively.

It is important to note that our findings may have limited generalizability and applicability beyond East Asian populations, given the similarity in genetic and environmental factors within this specific group. First, we were unable to adjust all confounding factors, due to the limited availability of GWAS data on East Asian ancestry, and the exclusion of certain SNPs associated with confounding factors such as BMI and fasting insulin levels due to screening criteria. Furthermore, despite our inclusion of a considerable number of cases in our study, the sample size for each individual cancer site remains limited. Particularly for pancreatic cancer, biliary tract cancer, ovarian cancer, and cervical cancer, the number of cases all did not exceed 1000. Therefore, in the future, it may be necessary to include a larger number of individuals from East Asian populations to further validate our research findings, once the SNP-outcome associations become available.

To summarize, our findings explored novel insights into the genetic correlation between T2D predisposition and decreased risks of several cancers (gastric cancer, breast cancer, esophageal cancer, colorectal cancer, hematological malignancy, lung cancer, prostate cancer and endometrial cancer), with the association attaining 80% statistical power specifically in gastric cancer, breast cancer, esophageal cancer, colorectal cancer and prostate cancer. But it’s needed to relize that carcinogenesis is a complex process, and it is affected by various external factors such as environmental factors, dietary factors, and lifestyle [28].

## Supplementary Information

Supplementary Material 1

Supplementary Material 2

Supplementary Material 3

Supplementary Material 4

Supplementary Material 5

Supplementary Material 6

Supplementary Material 7

Supplementary Material 8

Supplementary Material 9

## Data Availability

The datasets of T2D-associated SNPs used and/or analyzed the current study are available at AGEN consortium (https://blog.nus.edu.sg/agen/summary-statistics/t2d-2020/). The datasets of FG and GH-associated SNPs used and/or analyzed the current study are available at MAGIC consortium (https://magicinvestigators.org/). The datasets of cancers-associated SNPs used and/or analyzed the current study are available at JENGER (http://jenger.riken.jp/en/result). Should you require access to the summary-level data for the utilized SNPs in our study, we kindly ask that you contact the corresponding author with a reasonable request.
