# Supplementary material for: A Mendelian randomization study of type 2 diabetes and cancer risk in East Asians
Source: Cancer Cell Int. 2025 Aug 4;25:295. doi: 10.1186/s12935-025-03929-1 (PMC12320361; doi:10.1186/s12935-025-03929-1)
Supplement: Supplementary file 5 — Supplementary Material 5 [file 12935_2025_3929_MOESM5_ESM.docx]

Table s3: the removed SNPs

| The reason for the deletion of specific SNPs | Data sources of SNPs | Removed SNPs |
| --- | --- | --- |
| being palindromic with intermediate allele frequencies | T2D | rs10830963, rs11257657, rs256904, rs340875, rs4237150, rs4736999, rs4739515, rs475002, rs6885132, rs7090695, rs7210161, rs7900112, rs8037894, rs838720, rs9368194, rs988748 |
|  | HbA1c | rs10231021, rs10830963, rs13089972, rs2001945 |
|  | FG | rs10830963, rs12712928, rs830030 |
| horizontal pleiotropy in MR analysis between T2D and cancers | Gastric cancer | rs9350271，rs55734389 |
|  | breast cancer | rs76878791 |
|  | Esophageal cancer | rs1260326 |
|  | Lung cancer | rs9461650，rs3869115 |
|  | Prostate cancer | rs7051939 |
| horizontal pleiotropy in MR analysis between FG and cancers | Esophageal cancer | rs780093 |
| horizontal pleiotropy in MR analysis between HbA1c and cancers | Colorectal cancer | rs174559 |
|  | hepatocellular carcinoma | rs4980325 |
|  | Cervical cancer | rs857725，rs9909940 |
| horizontal pleiotropy in reverse MR analysis between T2D and cancers | breast cancer | rs3095316，rs4784227，rs73010941，rs939476 |
|  | Colorectal cancer | rs11066015, rs76681197 |
|  | Lung cancer | rs6937083, rs201414107 |
