# Supplementary material for: A Mendelian randomization study of type 2 diabetes and cancer risk in East Asians
Source: Cancer Cell Int. 2025 Aug 4;25:295. doi: 10.1186/s12935-025-03929-1 (PMC12320361; doi:10.1186/s12935-025-03929-1)
Supplement: Supplementary file 9 — Supplementary Material 9 [file 12935_2025_3929_MOESM9_ESM.docx]

**STROBE-MR Checklist**

| **​Section​** | **​Item​** | **​Article Reference​** | **​Page/Paragraph​** |
| --- | --- | --- | --- |
| ​**Title & Abstract**​ | 1. Title identifies the study as Mendelian randomization (MR) | Title includes "A Mendelian randomization study" | Title |
|  | 2. Abstract summarizes key elements (background, methods, results, conclusions) | Abstract includes study objectives, methods, key findings, and conclusions | Abstract |
| ​**Introduction**​ | 3. Scientific background and rationale for the study | Discusses gaps in T2D-cancer associations in East Asian populations | Introduction, Paragraphs 1-2 |
|  | 4. Justification for MR (avoiding confounding/reverse causation) | Highlights limitations of observational studies and advantages of MR | Introduction, Paragraph 3 |
| ​**Methods**​ | 5. Data sources (exposure, outcome, genetic instruments) | Data from JENGER, AGEN, and MAGIC consortia | Materials & Methods → *Sources of data* |
|  | 6. Criteria for selecting genetic instruments (p-value, LD, F-statistic) | p < 5×10⁻⁸, r² < 0.001, distance > 1 Mb, F-statistic > 10 | Materials & Methods → *Selection of instrumental variables* |
|  | 7. Strength of instrument-exposure associations (F-statistic) | Reports F-statistic > 10, excluding weak instruments | Same as Item 6 |
|  | 8. Statistical methods (MR models, sensitivity analyses) | IVW, MR-Egger, weighted median, MR-PRESSO, multivariable MR | Materials & Methods → *Statistical analysis* |
|  | 9. Methods to address pleiotropy/heterogeneity | Cochran’s Q test, MR-Egger intercept, MR-PRESSO outlier removal | Same as Item 8 |
|  | 10. Reverse MR analysis | Bidirectional MR for T2D and cancer | Materials & Methods → *Statistical analysis* |
| ​**Results**​ | 11. Sample sizes (exposure, outcome, number of instruments) | Lists case/control numbers and SNP counts (e.g., 174 T2D SNPs) | Materials & Methods → *Sources of data* / Results |
|  | 12. Instrument-exposure associations (β, SE, p-value) | Supplementary Tables S1-S3 provide SNP details | Supplementary Tables S1-S3 |
|  | 13. Primary MR results (OR, 95% CI, p-value) | Figure 2 shows T2D-cancer associations (e.g., gastric cancer OR = 0.875) | Results → *Fig. 2* / Supplementary Table S4 |
|  | 14. Sensitivity analyses (pleiotropy, heterogeneity) | MR-Egger intercept (p > 0.05), Cochran’s Q indicates heterogeneity | Results / Supplementary Table S5 |
|  | 15. Reverse MR results (if applicable) | Supplementary Tables S6-S7 report reverse MR results | Results / Supplementary Tables S6-S7 |
| ​**Discussion**​ | 16. Interpretation of results (consistency/divergence from prior studies) | Compares findings with European populations, discusses East Asian-specific factors | Discussion, Paragraphs 1-2 |
|  | 17. Limitations (instrument assumptions, sample size, confounding) | Notes unadjusted confounders (BMI/insulin), small sample size for some cancers | Discussion, Second-to-last paragraph |
|  | 18. Implications for clinical/public health practice | Emphasizes precision prevention strategies for East Asians | Discussion, Final paragraph |
| ​**Other Information**​ | 19. Data availability statement | Links to consortium data (AGEN, MAGIC, JENGER) | Declarations → *Availability of data and materials* |
|  | 20. Ethics approval and informed consent | States compliance with the Declaration of Helsinki and institutional ethics approval | Declarations → *Ethics approval and consent to participate* |
|  | 21. Funding and conflicts of interest | Lists grant numbers ,declares no conflicts | Declarations → *Funding* / *Competeting interests* |

**​**
